# Development of a quantitative multiparametric ultrasound and deep learning classifier for the detection of prostate cancer

**DOI:** 10.1007/s00330-026-12323-y

**Published:** 2026-01-30

**Authors:** Florian Delberghe, Xueting Li, Daniel L. van den Kroonenberg, Simona Turco, Wim Zwart, Giuseppe Valvano, Auke Jager, Arnoud W. Postema, Hessel Wijkstra, Jorg R. Oddens, Massimo Mischi

**Affiliations:** 1 Biomedical Diagnostics Lab, Department of Electrical Engineering, Eindhoven University of Technology, Groene Loper 3, 5612 AE Eindhoven, The Netherlands; 2 Amsterdam UMC, Department of Urology, Boelelaan, 1117 Amsterdam, The Netherlands; 3 Cancer Center Amsterdam, Boelelaan, 1117 Amsterdam, The Netherlands; 4 Angiogenesis Analytics, Den Bosch, The Netherlands; 5 Leiden University Medical Center, Department of Urology, Leiden, The Netherlands

**Keywords:** Prostate cancer, Multiparametric ultrasound, Contrast enhanced ultrasound, Shear wave elastography, Classification.

## Abstract

**Objectives:**

Prostate cancer (PCa) diagnosis is increasingly guided by imaging, with ultrasound (US) emerging as a cost-effective and widely accessible modality. This study develops a deep learning-based classifier predicting the presence of clinically significant (cs)PCa using quantitative features extracted from 3D multiparametric (mp)US.

**Materials and methods:**

A multicenter prospective cohort of 327 patients with suspicion of PCa underwent transrectal 3D mpUS scanning, including dynamic contrast-enhanced US and shear-wave elastography. Acquisitions were registered to 3D histology from radical prostatectomy, which served as the reference standard for the presence of csPCa. Voxels within lesions with International Society of Urological Pathology (ISUP) Grade Group ≥ 2 were considered malignant, and the rest were benign. A 3D deep learning classifier was trained on quantitative mpUS features to detect csPCa. The classifier was trained and internally evaluated on 250 patients and externally evaluated on 77 patients acquired later. Classifier performance was evaluated per voxel using the area under the receiver operating characteristic curve (ROC AUC).

**Results:**

Using quantitative mpUS features from 327 patients, the classifier achieved a ROC AUC of 0.87 (95% CI: 0.85–0.89) on the internal evaluation set, using 7-fold cross-validation. On the external evaluation cohort, the classifier achieved a ROC AUC of 0.88 (95% CI: 0.87–0.89).

**Conclusion:**

The proposed classifier accurately detects csPCa using quantitative features from 3D mpUS and generalizes well to the external dataset. These results support mpUS as a promising, cost-effective tool for csPCa diagnosis.

**Key Points:**

***Question:*** *Can quantitative features extracted from 3D multiparametric ultrasound (mpUS) reliably detect clinically significant prostate cancer (csPCa), enabling more accessible and affordable diagnosis?*

***Findings:*** *Predicting csPCa using quantitative multiparametric ultrasound features achieved an area under the receiver operating characteristic curve of 0.87, increasing to 0.88 when externally evaluated*.

***Clinical relevance:*** *Our proposed deep learning-based classifier using quantitative 3D mpUS features accurately detects csPCa, as validated on the largest mpUS prostate dataset to date. This opens the door to ultrasound as an accurate, cost-effective method for csPCa detection.*

**Graphical Abstract:**

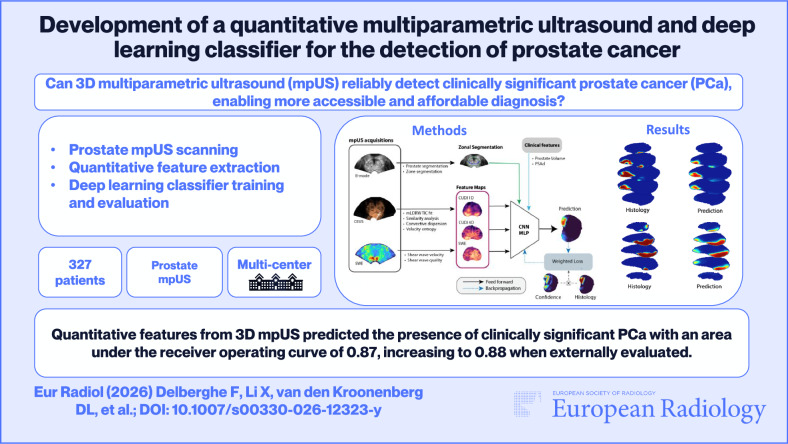

## Introduction

Prostate cancer (PCa) is one of the most prevalent malignancies in men. Early detection is crucial for effective treatment and improved survival rates. For decades, the standard of care for PCa was a transrectal ultrasound-guided (TRUS) systematic prostate biopsy, performed in patients with elevated prostate-specific antigen (PSA) levels or abnormal digital rectal examination [1]. However, this method carries risks of underdiagnosis and overtreatment [2, 3].

Recent advances in MRI have led to guidelines recommending pre-biopsy multiparametric (mp)MRI and mpMRI-targeted biopsy for men with suspected PCa [4]. Multiple studies have demonstrated that mpMRI-targeted biopsy improves the detection rate of clinically significant (cs)PCa while reducing the identification of indolent PCa compared to systematic biopsies alone [5, 6]. However, MRI has several limitations, including high costs, limited availability, and high inter-observer variability [7].

AI-driven computer-aided diagnostic (CAD) systems have emerged as a promising solution to address the challenge of inter-observer variability [8–12]. The PI-CAI study demonstrated that AI models, trained using thousands of patient examinations, are non-inferior to radiologists in detecting csPCa using mpMRI in a retrospective setting [13]. These results highlight the potential of AI-driven CAD systems in improving PCa diagnosis.

Although AI may reduce inter-observer variability in MRI interpretation, it does not address the challenges of high cost and limited availability [12]. These ongoing limitations highlight the need for alternative or complementary imaging modalities.

Multiparametric ultrasound (mpUS) has shown promise as an alternative to mpMRI for csPCa detection [14]. The CADMUS trial demonstrated that mpUS not only performs comparably to mpMRI but can also detect lesions missed by mpMRI [15]. While the CADMUS trial relied on the semi-quantitative interpretation of mpUS images, assigning Likert scores based on visual assessment of multiple US modalities, we propose to extract quantitative mpUS features. This shift from subjective scoring to objective and reproducible measurement enables the direct use of quantitative parameters in AI-driven CAD systems. Such a system automatically integrates complementary information from multiple imaging modalities, allowing the model to capture complex patterns associated with csPCa. This approach could provide a standardized, cost-effective pathway for csPCa detection, supporting both pre-biopsy triage and biopsy targeting while addressing key limitations of MRI.

This study focuses on the development and evaluation of an AI-driven CAD tool, based on 3D mpUS for the diagnosis of csPCa. Using radical prostatectomy (RP) specimens as the reference standard, the aim is to create a reliable algorithm to be used in a CAD system for csPCa diagnosis.

## Materials and methods

### Patient population

This study was approved by the institutional review board under reference number 2020_268#B202178. A total of 604 patients were enrolled in a prospective multicenter (four centers) trial conducted in the Netherlands between June 2021 and February 2024 (NCT04605276). The study includes both patients who underwent RP for biopsy-proven csPCa and patients with no or clinically insignificant PCa, who did not undergo RP. Absence of csPCa was determined by negative mpMRI (PI-RADS ≤ 2 [16]) and negative biopsy. All patients underwent 3D mpUS scanning, acquiring B-mode, CEUS, and SWE performed with a GEHC LOGIQ^™^ E10 US scanner and a RIC5-9D endocavitary probe (GE HealthCare)—acquisition details are available in the supplementary materials. During the acquisitions, the probe was held in a fixture to limit movement during and between scans [17].

**Fig. 1:**
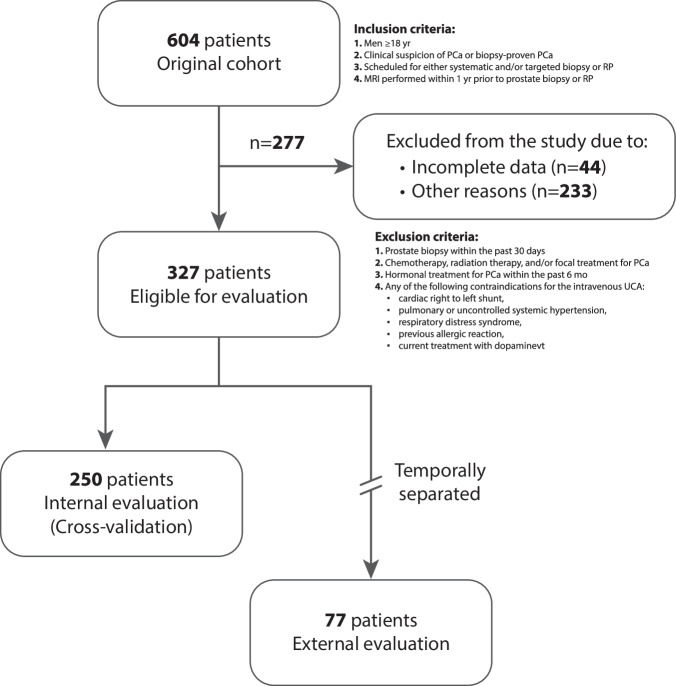
Workflow diagram of patient inclusion

We included and excluded patients according to the criteria in Fig. 1 (taken from Table 1 of Jager et al [18]). This left 327 patients with complete and usable data, including 29% of patients without csPCa—no RP and/or International Society of Urological Pathology (ISUP) grade group 1. Details of the acquisition protocol are available in the published study protocol [18].

**Table 1:** Patient clinicopathological characteristics (internal cohort *N *= 250, external cohort *N* = 77)

	Internal testing set	External testing set
Age: median [IQR]	69 [64–73]	69 [66–73]
Serum PSA (ng/mL): median [IQR]	8.0 [6.0–12]	8.1 [5.4–10]
US Prostate volume (cc): median [IQR]	45.2 [31.9–61.8]	45.2 [34.4–66.1]
PSA density: median [IQR]	0.18 [0.12–0.27]	0.16 [0.11–0.23]
Digital rectal exam: *N* (%)		
Benign	151 (60)	50 (65)
cT2	91 (37)	25 (31)
cT3/4	4 (2)	2 (2.6)
Missing	4 (2)	2 (2.6)
MRI PI-RADS score: *N* (%)**		
PI-RADS ≤ 2	69 (28)	41 (53)
PI-RADS 3	12 (5.2)	2 (2.6)
PI-RADS 4	76 (30)	11 (14)
PI-RADS 5	92 (37)	23 (30)
No MRI	1	0
Radiological tumor stage: *N* (%)**		
mT2	104 (42)	20 (26)
mT3a	48 (19)	13 (17)
mT3b	10 (4)	2 (3)
mT4	0	0
Not reported	88 (35)	41 (53)
ISUP grade from prostatectomy: *N* (%)**		
No RP	71 (28)	41 (53)
1	2 (1)	1 (1)
2	84 (34)	14 (18)
3	71 (28)	13 (17)
4	1	0
5	21 (8)	8 (10)

Statistical differences between the two cohorts were computed with a two-sided Mann–Whitney *U*-test for both continuous (age, PSA, prostate volume, PSA density) and ordinal variables (digital rectal exam, PI-RADS, tumor stage, ISUP grade). Note: Asterisks denote a significant difference between internal and external evaluation cohorts: ^**∗**^ *p* < 0.05, ^**∗∗**^ *p* < 0.01, ^**∗∗∗**^ *p* < 0.001

*PSA* prostate-specific antigen, *US* ultrasound,* ISUP* International Society of Urological Pathology

Patients were then divided into two groups: 250 patients to be used for training and internal evaluation, and 77 patients not included in the training to be used for external evaluation. The external evaluation group consisted of patients enrolled in the study once the performance of the algorithm—on the first group of 250 patients—had saturated. This temporal separation occurred during the trial, such that the participating centers and the acquisition protocol remained the same for both cohorts. Details of the groups are provided in Table 1.

### Clinical reference standard

The reference standard for the presence and location of csPCa was based on RP specimens. After RP, prostates were fixated and divided into 4 mm-thick slices for histopathological annotation (Fig. 4 in Jager et al [19]). All lesions with the ISUP grade group ≥ 2 were considered malignant, as defined by our clinical protocol [18, 20]; all prostate tissue outside those lesions was considered benign.

Subsequently, 3D reconstruction of the histology slices was performed [21], which was then registered to the 3D mpUS scan space [19]. Between histological slices, the probability of csPCa was calculated using Wiener interpolation [22] using the (assumed isotropic) autocorrelation from the clinical reference, evaluated on the histological slices. As a result, moving away from a region with known class membership makes the probability of a voxel being malignant approach a value corresponding to the prevalence of malignant voxels in the dataset, approximately 5%.

### Contrast-enhanced ultrasound features

#### Time intensity curve modeling

At every location in the prostate, the (linearized) scan intensity is proportional to the UCA concentration [23]. Approximating the UCA bolus as traveling along a tube from the point of injection to the prostate, the modified local density random walk model (mLDRW) can be used to estimate parameters that describe the time-intensity curves (TICs) [23]. Those parameters summarize the vascular dynamics from the site of bolus injection to the imaging location and are correlated with the presence of csPCa [24].

This solution to the convective dispersive process only accounts for the temporal dynamics of UCA concentration. It does so by assuming that at some point before the arrival in the prostate, the UCA bolus can be approximated as a Gaussian distribution in space. Parameters derived from the mLDRW model are referred to as 1D CUDI.

#### Convective dispersion

Instead of assuming the UCA starting distribution, it can be measured from the TICs, and the spatio-temporal solution to the 3D convective dispersion equation can be derived. Using the least squares method, the local blood velocity and diffusion coefficient (denoted as v and D, respectively) are estimated from neighboring TICs. The value of both those parameters has been shown to increase in the presence of PCa [25]. This group of parameters, along with others capturing both the spatial and temporal TIC information, is hereafter referred to as 4D CUDI.

#### Similarity analysis

Similarity between neighboring TICs also leverages spatio-temporal information. In a region where the microvasculature is more chaotic, TICs are more correlated [26], for example, due to the tortuosity of capillaries. This is quantified by the correlation between a TIC and its neighbors—in both the time and frequency domains—and the mutual information between TICs [26–28].

#### Velocity entropy

The final group of quantitative spatio-temporal CEUS features used in this study relates to the entropy of the blood velocity field. From the delay between the arrival times of the UCA in different locations, the magnitude and direction of the blood velocity can be estimated. Then, comparing the velocity at a given location with its surroundings, the entropy and conditional entropy of the blood velocity field can be estimated. Once more, the increased disorder in the microvasculature of cancerous tumors results in higher entropy of the velocity field [29].

### Shear-wave elastography features

In addition to vascular changes, PCa is associated with increased tissue stiffness [30], which can be quantitatively measured using shear-wave elastography (SWE). A motorized mechanism inside the probe enables 3D SWE by automated stepwise scans, capturing 25 planes from the base to the apex. Shear wave particle velocity signals were derived by the one-dimensional Loupas autocorrelator [31], and elasticity and quality maps were computed using the cross-correlation method between adjacent positions [32]. The resulting 2D maps were stacked and linearly interpolated to align with the histological ground truth to create 3D feature maps.

### Clinical features

The development of csPCa is often associated with an increased prostate volume and increased serum PSA relative to the prostate volume, known as PSA density (PSAd) [33, 34]. As these variables are predictive of the presence of csPCa, they were also included as an input to the classifier. Prostate volume was measured using an automated segmentation algorithm applied to the B-mode acquisitions [35], and PSAd was calculated from the prostate volume and the most recent PSA value.

### Classifier

#### Model architecture

**Fig. 2:**
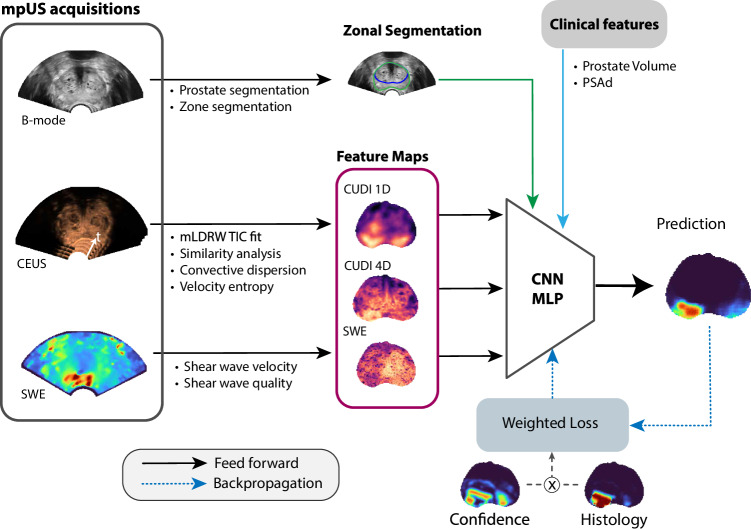
Schematic of the feature extraction, classification, and training procedures. The weighted binary cross-entropy loss function uses information about the confidence in the clinical reference. CNN, convolutional neural network; MLP, multi-layer perceptron; CUDI, contrast ultrasound dispersion imaging; SWE, shear wave elastography; PSAd, prostate-specific antigen density

**Fig. 3:**
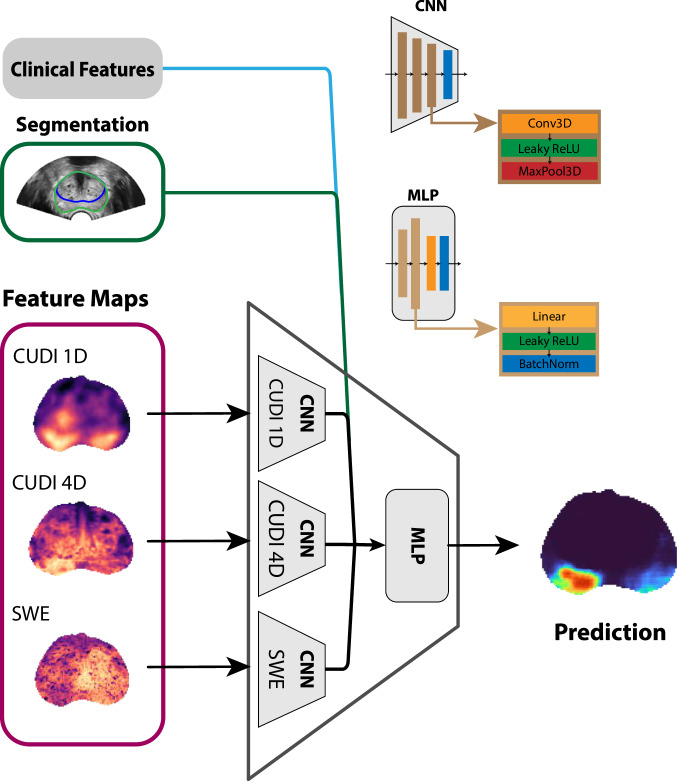
Detailed diagram of the CNN-MPL classifier architecture. CNN, convolutional neural network; MLP, multi-layer perceptron; CUDI, contrast ultrasound dispersion Imaging; SWE, shear wave elastography

**Fig. 4:**
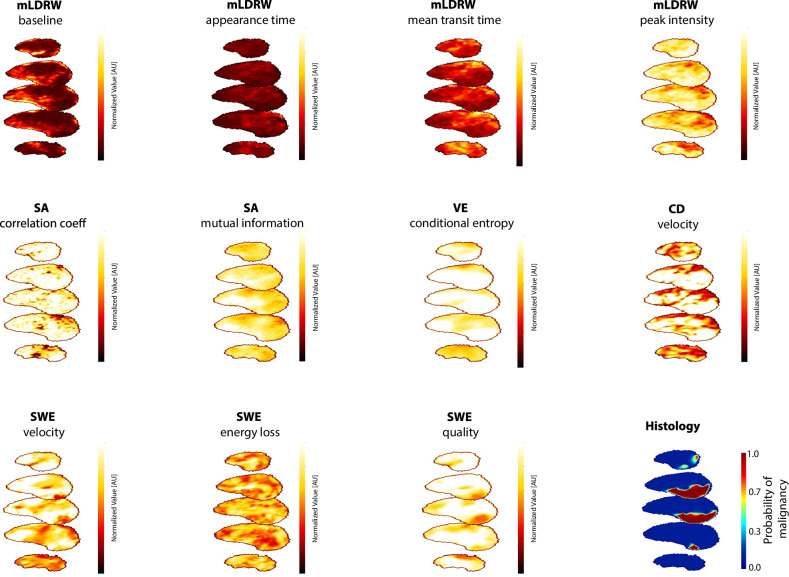
Transverse sections from the selected feature and histology maps for an example patient with csPCa. mLDRW, modified local density random walk; SA, similarity analysis; VE, velocity entropy; CD, convective dispersion; SWE, shear-wave elastography

The classifier used to predict the presence of csPCa is based on a 3D convolutional neural network (CNN)-multilayer perceptron (MLP) illustrated in Fig. 2 and Fig. 3. The CNN-MLP is composed of four subunits; three CNNs produce embeddings for each group of features (CUDI 1D, CUDI 4D, and SWE), followed by an MLP to combine the concatenated embeddings from the CNNs. The CNN-MLP contains 47k trainable parameters, takes dense 3D feature maps as input (Fig. 4), and outputs a dense 3D probability map indicating the likelihood of csPCa at each location in the prostate.

#### Classifier training

The classifier is trained using a 7-fold cross-validation scheme. To minimize biases between the folds, patients are divided into four subgroups based on the size of the csPCa lesions—no csPCa being one of the subgroups. The proportion of each subgroup is kept consistent across the folds. To improve the behavior of the gradients during backpropagation, each feature map is standardized using the mean and standard deviation calculated over the entire dataset. Feature selection was performed using forward selection with the area under the receiver operating characteristic curve (ROC AUC) as the evaluation metric. The CNNs for each feature group are pre-trained independently. During pre-training, to compensate for the class imbalance [36], each batch was generated with 50% positive samples by undersampling the majority class. As the lesions are small compared to the prostate, only 5% of the (approx. 10 M total) voxels are in malignant lesions. Later, all three CNNs are combined with the MLP connecting layer for fine-tuning. Fine-tuning was performed with a prevalence of 5%—matching the true prevalence in the dataset—and only the MLP was trained while the CNNs had their weights frozen. The lower fine-tuning prevalence, compared to the CNN training, did not influence performance, but it resulted in probability maps with improved visual quality.

Training was performed using the weighted cross-entropy loss and the Adam optimizer without regularization on the models’ weights. Data augmentation (additive white Gaussian noise and random offset) was used to limit overfitting and improve generalization. The model was trained for a maximum of 100 epochs and was stopped early if either the validation ROC AUC stopped improving or the training and validation losses did not change (both computed over 10 epochs).

As the registration process between the 3D pathology and the 3D mpUS scans leads to some inaccuracies [20], voxels, especially around the edges of lesions, may be misclassified. To prevent poisoning the classifier during training, the confidence that a voxel belongs to either class is used to weigh the binary cross-entropy loss function, illustrated in Fig. 2. The confidence is computed as ${w}_{{vox}}=\,\alpha /\left({\sigma }^{2}+\alpha \right)$, where $\alpha$ is a regularization constant ($\alpha$ = 0.2), ${\sigma }^{2}=p\,\left(1-p\right)$, and $p$ is the probability of a voxel to contain csPCa. One can think of the true class membership of a voxel being the result of a Bernoulli process with probability $p$ obtained from the Wiener interpolation of the RP reference. In that case, the uncertainty of the result (or entropy) is proportional to the variance of the distribution.

#### Control classifier

Because of attenuation and reduced resolution in the far field, the features can be location-dependent. As the peripheral zone (PZ) and transitional zone (TZ) of the prostate are located in similar locations across all TRUS scans (near and far-field, respectively) but differ in csPCa prevalence [37], the classifier may rely on this spurious correlation between the location of a voxel and the presence of csPCa.

To ensure that the discriminative power of the features is not reliant on the voxels’ location, a naïve classifier is used as a control. This control classifier is trained using only the location of voxels in the field of view and the zone to which they belong. The same location information is then fed to each CNN subunit to prevent re-learning this information from the feature maps. This way, the classifier can better leverage the discriminative power of the quantitative features.

#### Evaluation

All models were evaluated using the ROC AUC as the main performance metric. This facilitates comparison with other studies, as this is the most common metric, and it is theoretically invariant to the prevalence of the disease [38], which makes it a good estimate of performance on a different population—given that the distribution of the features remains the same. For each experiment, the cross-validation was repeated five times with a different random seed, allowing the estimation of the mean and standard deviation of the confidence-weighted ROC AUCs. In addition, given the conflicting results in the literature regarding the zone dependence of PCa classifiers [39–41], we also evaluated the performance of all our classifiers separately for the TZ and PZ.

## Results

**Fig. 5:**
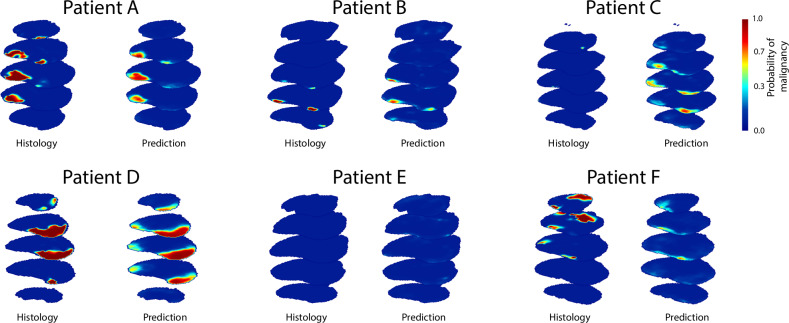
Transverse sections of 3D reconstructed histology and 3D prediction maps for four example patients with diverse csPCa lesion shapes. Patient B has multiple small lesions, while patient E has no csPCa. Patients C and D are examples of false positive and false negative predictions, respectively

On the internal evaluation set of 250 patients (median age: 69 years, interquartile range (IQR): 64–73), the proposed classifier achieved a ROC AUC of 0.870 (95% CI: 0.848–0.891) using all feature groups, as shown in Table 2. Using Youden’s J statistic [39], the optimal point on the ROC curve was found to be at 76.8% (95% CI: 64.0–89.5) sensitivity and 80.2% (95% CI: 71.8–88.6) specificity. Predicted csPCa probability maps for six example patients are shown in Fig. 5. The predicted csPCa locations closely match their true positions across diverse examples: small, large, multiple, distant, and even no lesions in patients A, B, C, and E. False positive and false negative examples are also shown for patients C and F, respectively.

**Table 2:** Area under the receiver operating curve for classifiers trained on different groups of features

Classifier feature group	Whole prostate		PZ		TZ	
	ROC AUC	95% CI	ROC AUC	95% CI	ROC AUC	95% CI
LOCATION	0.730	[0.725, 0.737]	0.656	[0.640, 0.672]	0.693	[0.664, 0.722]
SWE	0.821	[0.784, 0.859]	0.771	[0.756, 0.786]	0.825	[0.749, 0.901]
CUDI 1D	0.866	[0.840, 0.891]	0.830	[0.818, 0.842]	0.863	[0.813, 0.913]
CUDI 4D	0.855	[0.837, 0.873]	0.816	[0.806, 0.827]	0.864	[0.826, 0.902]
CUDI 1D + SWE	0.857	[0.828, 0.885]	0.823	[0.804, 0.841]	0.855	[0.795, 0.915]
CUDI 4D + SWE	0.862	[0.841, 0.884]	0.833	[0.816, 0.851]	0.855	[0.802, 0.907]
CUDI 1D + CUDI 4D	0.865	[0.844, 0.886]	0.834	[0.813, 0.855]	0.857	[0.809, 0.905]
ALL: Internal evaluation	0.870	[0.848, 0.891]	0.842	[0.826, 0.858]	0.862	[0.821, 0.903]
ALL: External evaluation	0.884	[0.876 0.893]	0.838	[0.829, 0.846]	0.884	[0.871, 0.897]

Reported values are evaluated in the whole prostate, in the peripheral zone (PZ) only, and in the transitional zone (TZ) only. The performance of the final classifier trained using all feature groups is reported on the internal and external evaluation sets. Confidence intervals are computed across repeated cross-validation folds

*CUDI* contrast ultrasound dispersion imaging, *SWE* shear wave elastography

The classifier generalizes well to the external evaluation set consisting of 77 patients (median age: 68 years, IQR: 66–73), achieving a ROC AUC of 0.884 (95% CI: 0.876–0.893). Despite the different demographics (53% of csPCa-negative patients versus 29%), the ROC AUC values did not significantly differ between the internal and external evaluation cohorts (two-sided paired *t*-test: *p* = 0.071).

After feature selection, the classifier used 8 CEUS-based features and 3 SWE-based features (detailed in Fig. 4), as well as 4 location features: the automated prostate and zone segmentation [35], the distance to the probe, and the scan elevation angle. Also selected were the prostate volume and PSAd (reported in Table 1).

As shown in Table 2, the control model, which used only the voxels’ location, demonstrates fair performance with a ROC AUC of 0.730 (95% CI: 0.725–0.737). Adding any of the three feature groups increases performance compared to the control model, with the 1D CUDI group achieving the best performance. Every combination of feature groups also outperformed each individual feature group on the whole prostate. However, the addition of the SWE group to 1D and 4D CUDI slightly decreased performance in the TZ. In addition, we observed that for all classifiers, the ROC AUC was higher in the TZ compared to the PZ.

## Discussion

This study demonstrates that quantitative 3D mpUS features extracted can reliably discriminate the presence of csPCa. These results are based on the largest reported mpUS dataset to date, using the most accurate clinical reference provided by 3D histology reconstruction from RP specimens. By combining features from all the US modalities, we achieved accurate detection of csPCa, with a voxel-wise ROC-AUC of 0.870 (95% CI: 0.848–0.891). Performance generalized well on an external evaluation set: ROC AUC of 0.884 (95% CI 0.874–0.892). This is an encouraging result, as the external evaluation cohort, like the biopsy-naïve population, has more csPCa-negative patients than the training cohort [42].

All quantitative US features exhibited discriminative value compared to naïve location-based classification. Moreover, all the feature groups have proven to be complementary despite the reported correlation between the temporal and spatiotemporal CUDI features [26]. Even though the quality of all features is expected to be lower in the TZ due to higher US attenuation in the far field, all classifiers performed best in this region. The lower prevalence of csPCa in the TZ could be skewing the evaluation [43].

The reported performance exceeds that of Wildeboer et al [44], which used similar features but a smaller patient cohort. While that study also employed 3D feature computation, only three transverse prostate planes were included in the analysis. In contrast, our study employed 3D histology reconstruction, which enables performance evaluation across the entire prostate, including the base and apex. Direct comparison to more studies is challenging, as most report performance after aggregating results into candidate regions, for example, surrounding the location of a biopsy needle [45]. Some studies have reported higher ROC AUC from aggregated regions than from voxel-level evaluation [44, 45], indicating that the voxel-level metrics could be a lower bound for potential performance using aggregated regions. With this assumption, our classifier outperforms other 3D mpUS studies in terms of ROC AUC [45–47].

As MRI is recommended for PCa diagnosis, it has led to numerous studies developing classifiers trained on MRI data, which was facilitated by the standardization of MRI acquisitions. The availability of large public datasets such as the PROSTATEx [48] and the PI-CAI [13] has further advanced CAD development for PCa detection. While the reported ROC AUC values reported in Saha et al [13] are higher compared to our study, they are based on scores aggregated over candidate lesions. Additionally, their classifier is advantaged by being an ensemble of the top five best-performing models from the PI-CAI challenge. If we, again, look at voxel-based performance as a lower bound for the region-aggregated performance, the results of our study fall within the range of the top five models (ROC AUC 0.861–0.909) [13]. These findings highlight 3D mpUS as an attractive alternative to MRI for csPCa detection.

While elasticity derived from SWE has been shown to be a strong predictor of csPCa [49, 50], SWE-derived features were the weakest predictors of csPCa in our study (Table 2). Adding the SWE group to the 1D and 4D CUDI features did not significantly improve performance in the whole prostate or the PZ, and slightly reduced performance in the TZ. We attribute this mainly to low SW quality, especially in the TZ. This degradation is likely caused by the absence of ultrafast tracking and the limited acquisition of only 25 planes per patient, leading to incomplete sampling of the prostate volume. As a result, missing data from interleaved tracking and out-of-plane information had to be interpolated, reducing the reliability of SWE-derived features. Furthermore, each plane was acquired using a single push event focused at one depth, resulting in strong attenuation of SWs in the far field, further degrading signal quality in the TZ. Similar limitations have been noted previously [51], highlighting the need for improved SWE acquisition protocols. Additionally, acoustic shadowing from the urethra and intrinsic biological differences between zones may further contribute to reduced performance in the TZ. Despite these limitations, SWE remains valuable, providing complementary information about tissue stiffness beyond CEUS. With improved acquisition strategies, SWE-derived parameters could substantially enhance model performance.

While the results are encouraging, the use of a single US system and UCA combination for all acquisitions may limit immediate clinical translation. Quantitative features, which reflect measurable quantities, should be independent of the imaging system; in practice, however, those features will be influenced by vendor-specific acquisition and image formation. In addition, the high fraction of RP patients in the training cohort introduces bias compared to the expected screening population [42]. This may require calibration, fine-tuning, and multi-vendor validation before wider use of this classifier across different scanners in multicenter cohorts.

Voxel-level performance does not directly translate to per-patient or lesion-aggregated performance, typically reported in clinical evaluations. To achieve a closer representation of potential clinical performance, the biopsy sampling procedure must be considered when calculating the confusion matrix. As this study focuses on voxel-level classifier performance, lesion-level evaluation was left to a companion study processing the dense probability maps into candidate lesions for targeted biopsy [52]. Ultimately, we aim to use our classifier in a head-to-head trial to evaluate the non-inferiority of mpUS-targeted biopsies compared to mpMRI-targeted biopsies on a representative biopsy-naïve population. The protocol for this head-to-head trial is described in van den Kroonenberg et al [53].

The proposed algorithm accurately detects the presence of csPCa and could serve as a powerful tool for pre-biopsy triage and biopsy targeting. While the use of multiparametric acquisition prevents real-time imaging, it still allows the prediction of candidate biopsy locations within minutes, making it fast enough to enable triage and targeted biopsy in the same clinical visit. As is needed for MRI-US fusion targeting, the output image of mpUS also must be fused with US biopsy imaging. If mpUS demonstrates non-inferiority to MRI in our current head-to-head trial, this would usher in mpUS as a competitive alternative to mpMRI that is cost-effective and potentially widely available.

As a final note, mpUS’s integration of complementary biomarkers, including echogenicity (from B-mode), (micro)vascular dynamics (from CEUS), and stiffness (from SWE), provides a wide assessment of tissue properties. The application of mpUS has been explored across various cancers, including breast [54], liver [55], and thyroid [56]. The flexibility of our AI-driven CAD approach, which extracts quantitative biomarkers from mpUS to predict the location of malignant lesions, could be readily adapted to these clinical scenarios. This highlights the potential of mpUS across diverse oncological applications.

In conclusion, this study describes a classifier capable of accurately predicting csPCa using quantitative 3D mpUS features. This classifier outperforms previous mpUS studies and approaches the performance of mpMRI-based methods. While the CNN-MLP-based classifier presented here was internally and externally evaluated using the largest reported mpUS datasets, with 3D histopathology as a reference, further work is needed to process the dense probability map into candidate lesions for targeted biopsy guidance and validate this method in a prospective manner. With the aggregation of the predicted csPCa location into candidate targeted biopsy locations, this classifier could serve as a powerful building block for a CAD system for PCa diagnosis that is both accessible and cost-effective.

## Supplementary information

Supplementary Material

## Abbreviations

CAD: Computer-aided diagnostic
CNN: Convolutional neural network
cs: Clinically significant
MLP: Multilayer perceptron
mp: Multiparametric
PCa: Prostate cancer
PSA: Prostate-specific antigen
PSAd: PSA density
PZ: Peripheral zone
ROC AUC: Area under the receiver operating characteristic curve
TRUS: Transrectal ultrasound-guided
TZ: Transitional zone
US: Ultrasound
