# Supplementary material for: Development of a quantitative multiparametric ultrasound and deep learning classifier for the detection of prostate cancer
Source: Eur Radiol. 2026 Jan 30;36(6):4539–50. doi: 10.1007/s00330-026-12323-y (PMC13212788; doi:10.1007/s00330-026-12323-y)
Supplement: Supplementary file 1 — Supplementary Material [file 330_2026_12323_MOESM1_ESM.pdf]

Development of a Quantitative Multiparametric Ultrasound and Deep Learning Classifier for the Detection of Prostate Cancer

ELECTRONIC SUPPLEMENTARY MATERIAL

Table S 1a: Settings for 4D Contrast Enhanced Ultrasound

Setting	Value
Axial voxel size (mm)	0.14 – 0.17
Dynamic range (db)	42
Gain (db)	55
Power level (%)	10
Transducer frequency (kHz)	3500
Mechanical index	0.10
Volume rate (Hz)	1.0
Radius start (mm)	12.389 – 15.2
Radius mean step size (mm)	0.14 – 0.17
Radius min step size (mm)	0.14 – 0.17
Radius max step size (mm)	0.14 – 0.17
Radius jitter (mm)	0.0
Azimuth range (deg)	110 – 150
Azimuth mean step size (mm)	0.79 – 0.81
Azimuth min step size (mm)	0.79 – 0.81
Azimuth max step size (mm)	0.79 – 0.81
Azimuth jitter (mm)	0.0
Elevation range (deg)	118 – 120
Elevation mean step size (mm)	2.30 – 2.372
Elevation min step size (mm)	2.30 – 2.372
Elevation max step size (mm)	2.30 – 2.372
Elevation jitter (mm)	0.0

Table S 1b: Settings for B-Mode

**Setting**	**Value**
Axial voxel size (mm)	0.05 – 0.14
Dynamic range (db)	69
Gain (db)	55
Power level (%)	100
Transducer frequency (kHz)	9000
Mechanical index	1.30
Radius start (mm)	12.389 – 15.2
Radius mean step size (mm)	0.05 – 0.14
Radius min step size (mm)	0.05 – 0.14
Radius max step size (mm)	0.05 – 0.14
Radius jitter (mm)	0.0
Azimuth range (deg)	110 – 150
Azimuth mean step size (mm)	0.30 – 0.32
Azimuth min step size (mm)	0.30 – 0.32
Azimuth max step size (mm)	0.30 – 0.32
Azimuth jitter (mm)	0.0
Elevation range (deg)	118 – 122
Elevation mean step size (mm)	0.70 – 0.72
Elevation min step size (mm)	0.70 – 0.72
Elevation max step size (mm)	0.70 – 0.72
Elevation jitter (mm)	0.0

Table S 1c: Settings for shear wave elastography

Setting	Value
Axial voxel size (mm)	0.09 – 0.10766
Dynamic range (db)	20
Gain (db)	14
Power level (%)	100
Transducer frequency (kHz)	9000
Mechanical index	1.4
Radius start (mm)	12.389 – 15.2
Radius mean step size (mm)	0.09 – 0.108
Radius min step size (mm)	0.09 – 0.108
Radius max step size (mm)	0.09 – 0.108
Radius jitter (mm)	0.0
Azimuth range (deg)	110 – 150
Azimuth mean step size (mm)	0.68 – 0.80
Azimuth min step size (mm)	0.68 – 0.80
Azimuth max step size (mm)	0.68 – 0.80
Azimuth jitter (mm)	0.0
Elevation range (deg)	119 – 121
Elevation mean step size (mm)	4.9 – 5.1
Elevation min step size (mm)	4.9 – 5.1
Elevation max step size (mm)	4.9 – 5.1
Elevation jitter (mm)	0.0
